# Sensing Traffic Density Combining V2V and V2I Wireless Communications

**DOI:** 10.3390/s151229889

**Published:** 2015-12-16

**Authors:** Julio A. Sanguesa, Javier Barrachina, Manuel Fogue, Piedad Garrido, Francisco J. Martinez, Juan-Carlos Cano, Carlos T. Calafate, Pietro Manzoni

**Affiliations:** 1 DIIS, University of Zaragoza, Ciudad Escolar s/n, Teruel 44003, Spain; jsanguesa@unizar.es (J.A.S.); jbarrachina@unizar.es (J.B.); mfogue@unizar.es (M.F.); piedad@unizar.es (P.G.); 2 DISCA, Universitat Politècnica de València, Camino de Vera s/n, Valencia 46022, Spain; jucano@disca.upv.es (J.-C.C.); calafate@disca.upv.es (C.T.C.); pmanzoni@disca.upv.es (P.M.)

**Keywords:** VANETs, vehicular networks, road side unit, density estimation

## Abstract

Wireless technologies are making the development of new applications and services in vehicular environments possible since they enable mobile communication between vehicles (V2V), as well as communication between vehicles and infrastructure nodes (V2I). Usually, V2V communications are dedicated to the transmission of small messages mainly focused on improving traffic safety. Instead, V2I communications allow users to access the Internet and benefit from higher level applications. The combination of both V2V and V2I, known as V2X communications, can increase the benefits even further, thereby making intelligent transportation systems (ITS) a reality. In this paper, we introduce V2X-d, a novel architecture specially designed to estimate traffic density on the road. In particular, V2X-d exploits the combination of V2V and V2I communications. Our approach is based on the information gathered by sensors (*i.e*., vehicles and road side units (RSUs)) and the characteristics of the roadmap topology to accurately make an estimation of the instant vehicle density. The combination of both mechanisms improves the accuracy and coverage area of the data gathered, while increasing the robustness and fault tolerance of the overall approach, e.g., using the information offered by V2V communications to provide additional density information in areas where RSUs are scarce or malfunctioning. By using our collaborative sensing scheme, future ITS solutions will be able to establish adequate dissemination protocols or to apply more efficient traffic congestion reduction policies, since they will be aware of the instantaneous density of vehicles.

## 1. Introduction

Intelligent transportation systems (ITS) are emerging as the solution to adequately control and manage information on the roads, offering drivers and passengers a plethora of new services focused on improving driving, especially those related to safety and collaborative driving. Modern ITS will completely change the way people access applications and services related to traffic, increasing the driving efficiency and the comfort of drivers and passengers [1].

The confluence of ubiquitous computing, wireless telecommunication, sensing capabilities and transportation technologies is extending the possibilities of the roads and highways. As for transportation, the traffic of our roads is undergoing a severe increase, and traffic congestion is currently one of the most important problems, especially in the most important cities around the globe. More specifically, traffic jams: (1) increase travel time, air pollution and fuel consumption; (2) deteriorate the transportation infrastructure; and (3) increase user frustration and enervation [2]. Traditionally, the density of vehicles has been used as one of the key parameters for evaluating the traffic state. A high density, in general, is associated with overcrowded traffic. Nevertheless, in urban environments, the vehicle density usually varies depending on the time of the day and the area considered (downtown, rush hours, *etc*.). Hence, getting accurate information about traffic density is crucial to better apply traffic congestion avoidance strategies to reduce contamination and to improve the traffic flow.

Vehicular networks (VNs) support all of the communications required to enable cooperative driving between vehicles and infrastructure nodes [3,4,5]. VNs comprise vehicle to vehicle (V2V) [6,7,8] and vehicle to infrastructure (V2I) [9,10] communications and have been widely studied by academia, public institutions and the industry, especially due to the new attractive services and applications that can be provided. In particular, VNs provide a wide range of applications related to managing traffic flow [11,12], network security [13,14], health matters [15], environmental protection [16], mobile infotainment [17,18], *etc*., although increasing drivers’ safety has been the main objective for the deployment of this kind of network [19,20]. The majority of these services will benefit from accurate traffic density awareness, since new adaptive dissemination mechanisms and more intelligent traffic management strategies can be used [21].

So far, several separate V2V or V2I-based approaches have been presented, although we consider that using them together could improve the system’s performance by adding new advantages. Hence, in this work, we present a V2X architecture that uses wireless sensors and combines V2V and V2I communications to make an accurate traffic density estimation. Unlike existing proposals, our architecture includes the advantages of V2I (macroscopic vision, global information, *etc*.) with the benefits of V2V (microscopic approach, accuracy, locality, *etc*.). More specifically, our approach considers both the beacons received per road side unit (RSU) (V2I) and per vehicle (V2V), as well as the main topology characteristics, to get an accurate estimation of the density of vehicles. According to our proposal, more efficient dissemination protocols can be used, and smarter traffic congestion reduction strategies can be applied. Additionally, our proposal provides fault tolerance capabilities, since it can continue operating despite any RSU failures.

This work is organized as follows: Section 2 presents the related work on density estimation solutions and fault tolerance schemes in vehicular environments. In Section 3, we introduce our V2X-density (V2X-d) architecture. Section 4 details the V2X real-time vehicle density estimation system. In Section 5, we assess the proposed solution and examine the results obtained. Finally, Section 6 concludes this paper.

## 2. Related Work

Although determining the density of vehicles is very important to better support useful applications in vehicular networks (e.g., improving communication capabilities or reducing vehicle congestion), only a few studies have studied how to estimate the density of vehicles to enhance wireless communications or improve the traffic flow. Additionally, few works have focused on the combination of V2V and V2I communications to enhance previously proposed applications or to overcome infrastructure failures without compromising reliability. Next, we present the most relevant works in these fields.

Regarding vehicle density estimation, the majority of works relied on infrastructure-based traffic information systems. Tyagi *et al.* [2] proposed a vehicle density estimation system that used the information gathered by roadside-installed microphones. More specifically, they used the cumulative acoustic signal comprising noise signals, such as engine noises, occasional honks, tire noises, engine-idling noises and air turbulence noises. However, this approach involves the deployment of microphones in all of the streets to be able to estimate the vehicle density. Tan and Chen [22] presented a system able to determine the traffic density state in a specific region of interest (ROI). In particular, their approach analyzes the captured video by combining AutoClass, an unsupervised clustering scheme, with hidden Markov models (HMMs). As expected, the deployment of video cameras in all of the streets is required to estimate the vehicle density. Additionally, this scheme involves a huge computational cost to analyze all of the videos. Maslekar *et al*. [21] proposed a direction-based clustering algorithm able to estimate the density of vehicles. Their algorithm also included a cluster-head switching mechanism. Simulation results demonstrated that their proposed algorithm accurately determined the vehicle density. However, a stable cluster within a vehicular framework is hard to implement, especially due to the high mobility of vehicles.

As shown, the majority of vehicle density estimation proposals have been designed to be implemented with a particular infrastructure, which usually requires the placement of traffic surveillance cameras, roadside-installed microphones or inductive loop detectors [22,23]. Nevertheless, all of these schemes can only determine the density of vehicles in *a priori* chosen areas (*i.e*., where these devices were deployed), making it nearly impossible to accurately estimate the density of vehicles within very large areas. Additionally, some proposals cannot provide accurate real-time density estimations, since they involve complex data processing and analysis. Thus, we consider that the combination of V2I and V2V-based communications can effectively overcome all of these issues.

Regarding the use of V2V communications to estimate vehicle densities, few works have been presented so far. Stanica *et al*. [24] presented a system able to adjust the minimum contention window according to the local vehicle density, thus improving the overall performance of 802.11. Additionally, they performed realistic simulations to compare different schemes able to estimate the local density in vehicular environments. More specifically, they presented the advantages and the limitations of each of them. Sanguesa *et al*. [25] presented a traffic density estimation system, which uses V2V communications to enhance the warning message dissemination in urban environments. Simulation results showed that their proposal accurately estimates the density of vehicles, thus supporting more efficient broadcast protocols.

As shown in previous proposals, each vehicle can determine the number of vehicles in its neighborhood. However, unlike our proposal, vehicles are only able to obtain density information in their surroundings, while being unable to determine the vehicle density of the complete scenario. Therefore, vehicles cannot accurately avoid traffic jams or determine the best route to travel to their destination. We consider that this issue could be solved by including some infrastructure nodes and thus combining the traffic information obtained by them with the information offered by vehicles to provide a global and more accurate perspective.

Other approaches are based on floating car data (FCD) transmission over cellular networks. In particular, these solutions gather the information produced by vehicles and upload it to control centers in charge of information processing and analysis [26]. Hence, control centers can also provide an estimation of the traffic density. In fact, FCD approaches have been used to gather information about real-time traffic conditions from a set of vehicles [27]. Other authors extended the FCD concept to the use of RSUs [28]. However, FCD-based approaches are more intimately associated with the use of cellular networks as the support infrastructure. Despite the benefits of these kinds of systems, they also present some drawbacks, such as communication fees and infrastructure overcrowding.

Although combining both V2I and V2V-based communications seems to be very promising to improve intelligent transportation systems, only a few works have been proposed so far. Torres *et al*. [29] studied the feasibility of using V2V and V2I communications to broadcast a video stream from the accident location to the road traffic authority. Their proposal additionally considers vehicles as data relays, meaning that drivers can directly be provided with a clear view of the accident, improving the traffic flow and helping them to better react to dangerous situations. The authors also provided a simulation analysis, including a comparison of different dissemination mechanisms specially designed for wireless networks. The results obtained showed the feasibility of their proposal, especially in highways with medium and high vehicle densities, although the authors stated the necessity of proposing more suitable schemes in such environments. Knapik *et al*. [30] presented several tips to avoid vehicular crimes. In particular, they proposed the electronic decal, a security function based on V2X communications able to significantly reduce car theft rates. Moreover, they provided a solution to integrate their approach into the message format proposed by the European Telecommunications Standards Institute (ETSI). Miller [31] proposed a V2X architecture. In particular, he called it V2V2I, and it enhanced the performance of traditional V2I-based architectures in terms of fast queries and responses. In his system, only super vehicles are allowed to maintain communication with the central station or with other super vehicles; the rest of the vehicles are only allowed to communicate with the super vehicle in charge of the area to which they belong at a given time. Experiments evaluated the results obtained when varying the number and size of zones; additionally, the author presented the advantages of the V2V2I architecture compared to V2I or V2V-based architectures. Miller claimed that his proposal integrates the benefits of both V2V and V2I architectures, that is to say, the fault tolerance operation of V2V-based approaches and the accuracy and fast queries provided by V2I-based architectures. However, unlike our proposal, no results were provided to demonstrate the fault tolerant assumption.

Overall, different approaches with the main goal of estimating vehicle density have been proposed. Additionally, other works have studied the combination of V2V and V2I to improve communication capabilities or increase traffic safety and security. However, to the best of our knowledge, still no work has addressed the use of both V2V and V2I communications to accurately and reliably estimate the density of vehicles in a given area.

## 3. The V2X-d Architecture for Real-Time Accurate Vehicle Density Estimation

In this work, we propose the use of both V2V and V2I communications (V2X) for vehicular density estimation. The V2X-d architecture is a novel approach specially designed to perform such estimations accurately and in real-time, taking advantage of the communication capabilities among vehicles and RSUs. In particular, our proposal is based on the information gathered by vehicles and RSUs, as well as the main topology characteristics, to give an estimation of instant vehicle density. Our approach can provide useful information to traffic management systems, allowing them to predict and anticipate alternatives to mitigate traffic jams.

**Figure 1:**
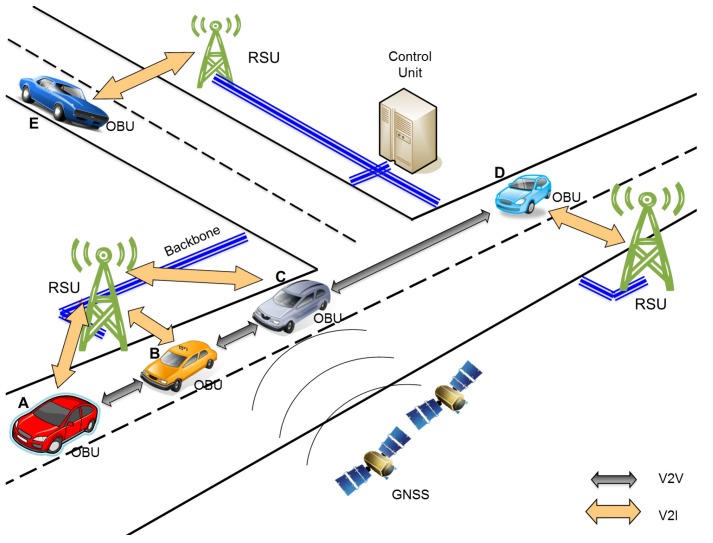
Combined V2V and vehicle-to-infrastructure (V2I) communication (V2X)-based vehicular density estimation architecture.

Figure 1 presents a general outlook of the V2X-d architecture. As depicted, all of the vehicles include an on-board unit (OBU) providing wireless communication capabilities. In particular, the OBU offers the required interfaces to carry out the vehicle density estimation according to the information gathered through V2V communications. Additionally, OBUs will obtain positioning information by accessing global navigation satellite systems (GNSS), e.g., the Global Positioning System (GPS) [32], or Galileo [33]. This information will be crucial to correctly select the areas of interest of the vehicle density estimation or to enable future density estimations by anticipating the vehicles’ movements. The implementation of the V2I side of our system requires the deployment of infrastructure equipment, *i.e*., RSUs. In particular, RSUs will help vehicles by providing them new services. The deployment of RSUs also provides communication capabilities among vehicles located in different areas, through a common backbone [34].

Our proposed architecture is similar to a previous one proposed by Bellalta *et al*. [35]. The main difference is that the authors considered wireless communication between RSUs, whereas in our proposed architecture, RSUs should benefit from wired connections. With this premise in mind, we try to avoid connectivity problems between RSUs that could appear in urban scenarios due to the presence of buildings, other obstacles or the distance between RSUs.

To acquire an accurate vehicular density estimation approach, we must first implement a system able to estimate the vehicle density by accounting for the information gathered by both V2I and V2V communications. Additionally, the system will be able to provide accurate results for every type of scenario. More specifically, our density estimation scheme should be automatically adjusted to the specific characteristics of the topology considered.

Using V2X-d, the limitations of the V2V or V2I-based systems can be overcome by combining the two approaches. In particular, vehicles can be aware of the number of vehicles in their surroundings, whereas RSUs can complement their information about traffic distribution through the density feedback provided by vehicles. Additionally, our proposal provides fault tolerance capabilities, since it can still provide accurate vehicle density estimations when some RSUs fail.

### 3.1. V2X-d Applications

The V2X-d architecture allows the development of useful applications oriented to transportation systems. As referred to above, the combination of both V2V and V2I communications can provide additional benefits towards reducing some problems or deficiencies observed when using these communication approaches separately. Next, we enumerate some of the most relevant.

**Figure 2:**
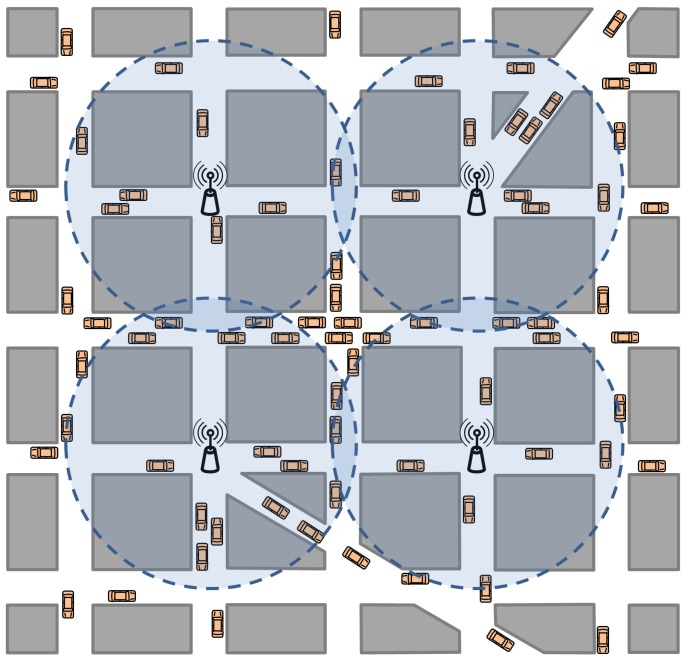
Example of blind spots due to insufficient RSU coverage.

- Gathering data related to the most visited zones, e.g., with the objective of improving the road preservation policies or enhancing the management of the most congested areas: All of this information can help authorities to decide future actions, improving the efficiency of transportation systems and reducing transportation costs. This is a key point since transportation issues highly affect the productivity and economic growth of developed countries.
- Broadcast storm reduction: Message delivery schemes are greatly affected by blind message broadcasting, which can provoke congestion and contention in the wireless channel, as well as massive packet collisions, drastically reducing communication performance [36]. In order to mitigate channel saturation, information about current traffic density can be used to determine how many vehicles are competing for the channel, thus allowing the use of corrective mechanisms, such as limiting non-critical information transmission. In [37], we demonstrated that broadcast storms can be mitigated or avoided when accounting for the current vehicle density in warning message dissemination schemes.
- Reducing traffic jams and pollution. The information collected by the RSUs deployed in a target area provides global knowledge of the scenario. Hence, areas with a high density of vehicles where traffic jams are likely to occur can be detected. This information would be forwarded to the vehicles in the scenario, allowing incoming vehicles to avoid problematic routes and reaching their destinations in a shorter time.
- Reducing arrival time for emergency services in the event of an accident, enhancing routes for emergency vehicles selecting streets with a low density of vehicles or changing the routes of other vehicles to facilitate the mobility of emergency vehicles: In [38], different solutions were proposed to efficiently redirect traffic, thus obtaining a short arrival time for emergency services and alleviating the occurrence of traffic jams when an accident takes place.
- Increasing the accuracy when estimating vehicle density if the required information cannot be collected by vehicles or RSUs separately: Depending on the particular deployment of RSUs in a scenario, blind areas unreachable by infrastructure units may appear, with the consequent reduction of accuracy when estimating the global density of vehicles. Figure 2 shows an RSU deployment example intended to cover a specific area with a high vehicle density and presenting some blind spots outside the reach of existing RSUs (e.g., one in the center of the scenario and others in the corners). This effect provokes differences between the real and the estimated vehicle densities in the scenario. Nevertheless, the information gathered by using V2V communications can be used by the RSUs to complete the information they are missing (*i.e*., adjusting their density estimations).

### 3.2. Qualitative Comparison against Other Density Estimation Approaches

Table 1 shows the main features of different existing schemes for vehicle density estimation. Studying the deficiencies of these schemes, we can conclude that the only approach that is able to fulfill all of the desired capabilities demanded by modern ITS services and traffic control authorities is the proposed V2X-d approach.

Environmental conditions, such as reduced light or adverse weather, greatly affect traditional methods based on infrastructure, e.g., ambient microphones or surveillance cameras. Inductive loop detectors are not affected by these problems, but the covered area where they are able to estimate vehicle density is limited to the streets where the data acquisition devices are installed, similarly to previous approaches. In addition, they lack fault tolerance, and broadcast storm mitigation techniques cannot be applied, since they are not able to warn vehicles to change their dissemination policies.

Density estimation schemes based on V2I communication provide good capabilities in terms of control of traffic congestion. However, their functionality regarding broadcast storm reduction and fault tolerance is limited. For example, in the event of an RSU malfunction, the density information of its covered area could not be used for density estimation, since these data would not be available. The potential failures of this system (damaged infrastructure devices, backbone errors, *etc*.) can be overcome by using our proposed V2X-d architecture. Combining V2V and V2I communications allows reducing the uncertainty of the information gathered and makes it as complete as possible, thereby improving the overall system in terms of fault tolerant capabilities.

Most of these problems can also be solved using V2V approaches, but the local knowledge they provide does not allow computing optimal vehicle routes. In particular, the information obtained by the vehicles is limited to their neighboring vehicles, and the available density estimation is also limited to their one-hop coverage area. The proposed V2X-d architecture incorporates the advantages of V2V and V2I approaches, granting the possibility of deploying new services for better traffic control and reduced travel time and vehicle emissions. Authorities, transport agencies and drivers will be able to take advantage of these features to make use of faster and better wireless communications.

**Table 1:** Qualitative comparison of density estimation schemes.

Feature	Cameras	Loop Detectors	Microphones	V2I	V2V	V2X-d
24/7 availability	✖	✔	✔	✔	✔	✔
Different light conditions	✖	✔	✔	✔	✔	✔
All-sound conditions	✔	✔	✖	✔	✔	✔
All-weather conditions	✖	✔	✖	✔	✔	✔
Real-time estimation	✖	✔	✔	✔	✔	✔
Wide coverage	✖	✖	✖	✔	✖	✔
Traffic jam avoidance	✔	✔	✔	✔	✖	✔
Broadcast storm mitigation	✖	✖	✖	✖	✔	✔
Fault tolerant	✖	✖	✖	✖	✔	✔

## 4. V2X-d Density Estimation Approach

In this section, the V2X-d vehicular density estimation mechanism is presented. As stated above, our approach relies on V2V and V2I communications with the objective of estimating the number of vehicles within a particular area in real-time.

In our vehicle density estimation system, a warning message dissemination scheme is considered, where vehicles periodically broadcast data about themselves or about anomalous situations, such as traffic jams or slippery roads. Our simulations include the possibility of vehicles sharing accident warning messages to increase the realism of the collected results. Specifically, two different operation modes are considered in our simulations for the involved vehicles: (i) warning mode; and (ii) normal mode. Vehicles in warning mode send warning messages to alert other vehicles about their status with a frequency of one message per second, thereby increasing the probability of channel congestion. Normal mode vehicles are in charge of rebroadcasting these warning messages, and additionally, they send periodic beacons with useful data about their position, speed, and so on. These beacons are sent every second, although they are not rebroadcasted by the rest of the vehicles.

Our density estimation system relies on simulation, since the evaluation of the proposed scheme requires a precise representation of: (i) the mobility of the vehicles; and (ii) multi-hop broadcast communications in urban environments. Two estimation functions are needed (*i.e*., the V2I and V2V-based functions), since different types of ITS services require using different information provided by each of them. For example, in order to reduce broadcast storms and traffic overhead in VANETs, only the local density of vehicles in the neighborhood of each vehicle needs to be determined. However, the systems designed to reduce traffic jams need to reroute vehicles to areas with lower traffic density, and so, they require global density information about the entire target area. Next, we present them in detail.

### 4.1. V2I-Based Density Estimation

An extensive simulation study was performed so as to propose a V2I-based approach to estimate vehicle densities with remarkable accuracy. The simulations included scenarios where the vehicle density was previously known, and the number of beacons received by each infrastructure unit during periods of T seconds was computed. It is noteworthy that vehicles transmit one beacon per second, and these packets are not forwarded by receiver vehicles, as opposed to warning messages. A time period of 30 s was considered, since the results presented in [25] showed that this value is optimal when compared to the results obtained using different time periods, in particular, 10, 20, 30, 60, 120 and 180 s.

The data collected was incorporated into a regression analysis process by using ZunZun [39]. This tool evaluates different prototypes of functions, such as polynomial, exponential and logarithmic, to achieve the best fit to the provided data obtained through our simulations. Using the relative error to discriminate among the different functions, Equation (1) was selected as the density estimation function based on V2I communications, since it provided the smallest error. The goal of this function is to determine the amount of vehicles per km${}^{2}$ in urban scenarios using as parameters the mean number of beacons received by each RSU, and the ratio between streets and junctions of the studied map, known as the SJ ratio or *SJR*.

(1) $$\begin{array}{c} f\left(B_{RSU},SJR\right)=a+b\cdot ln\left(B_{RSU}\right)+\frac{c}{SJR}+d\cdot ln{\left(B_{RSU}\right)}^{2}+\\ +\frac{f}{SJR^{2}}+\frac{g\cdot ln(B_{RSU})}{SJR} \end{array}$$

In this equation, the $B_{RSU}$ value represents the mean number of beacons received per RSU, and the SJR value is computed as the streets/junctions ratio from the urban map where the transmissions take place. The specific values of the different coefficients provided by ZunZun (a,b,c,d,f,andg) are shown in Table 2.

**Table 2:** V2I equation coefficients.

Coeff.	Value
a	2.304E+02
b	1.907E+01
c	−4.295E+02
d	3.188E+01
f	1.880E+02
g	−6.813E+01

We evaluated the accuracy of the proposed function measuring the error in the density estimation. Table 3 contains different measures of error made by the density estimation function compared to the actual values retrieved during the simulations. It is noticeable that the mean relative error is as low as 3.04%.

**Table 3:** V2I density estimation error.

Error	Absolute	Relative
Minimum	−5.399E+01	−1.226E+00
Maximum	4.837E+01	1.698E+00
Mean	2.848E−13	3.041E−02
Std.Error of Mean	2.422E+00	3.544E−02
Median	2.372E−01	1.583E−03

### 4.2. V2V-Based Density Estimation

After determining the function having the best fit using V2I-based information, we similarly obtained a function designed to estimate the vehicle density in a particular area based on the information collected by the vehicles and accounting for the complexity of the roadmap. However, in this case, our estimation approach relies on the number of neighbors (instead of the amount of beacons received), since this scheme is able to characterize the mobility of vehicles more accurately. Additionally, our initial experiments demonstrated that, using the number of neighbors as input, is able to provide more precise results than using the number of beacons in V2V communications.

We call neighbors to those vehicles those that are reachable by one-hop messages, without requiring any additional rebroadcast, *i.e*., they are within radio range, not being blocked by any obstacle, such as buildings, and assuming symmetry in the wireless communication links. In our system, all of the vehicles maintain a neighbor list that is built by using the beacons exchanged periodically by vehicles, avoiding any additional channel overhead. Whenever a new beacon is received, each vehicle checks its neighbor list to determine if the sender is a new neighbor, thereby adding this vehicle to the list. The neighbors’ list is also updated when new beacons are not received from a former neighbor after 2 s. In that case, the neighbor is removed from the list.

Different functions like logarithmic, exponential, and so on, were again tested using the data collected from our simulations. The regression analysis performed determined that a polynomial equation is able to offer the best fit to the experimental data. Equation (2) presents the vehicular density estimation function that uses the number of neighbors and the SJ ratio of the scenario as inputs, providing the calculation of the number of vehicles per km${}^{2}$ in the area as a result.

(2) $$\begin{array}{c} f\left(Neighb,SJR\right)=a+b\cdot Neighb+c\cdot SJR+d\cdot Neighb^{2}+f\cdot SJR^{2}+\\ +g\cdot Neighb^{3}+h\cdot SJR^{3}+i\cdot Neighb\cdot SJR+j\cdot Neighb^{2}\cdot SJR+\\ +k\cdot Neighb\cdot SJR^{2} \end{array}$$

In this equation, Neighb is the number of neighbors and SJR is the streets/junctions ratio of the road scenario. Table 4 lists the specific values obtained for coefficients of the polynomic function (a,b,c,d,f,g,h,i,j, and k).

**Table 4:** V2V equation coefficients.

Coeff.	Value
a	−7.917E+02
b	−6.599E−01
c	2.272E+03
d	1.199E+00
f	−2.102E+03
g	−1.751E−02
h	6.310E+02
i	−4.811E+00
j	−7.644E−01
k	1.460E+01

**Table 5:** V2V density estimation error.

Error	Absolute	Relative
Minimum	−2.490E+01	−1.446E−01
Maximum	2.546E+01	1.972E−01
Mean	6.634E−13	4.147E−03
Std. Error of Mean	1.142E+00	9.005−03
Median	1.414E−01	2.183E−03

The accuracy of our proposed function was tested by means of error measurements in the estimations. Table 5 presents different types of errors and their corresponding values for the proposed density estimation function in comparison to the actual values. In this case, the relative error is reduced to 0.41%.

As expected, higher estimation errors appear when the V2I-based density estimation approach is used instead of the V2V-based approach, since the latter represents a more fine-grained option (*i.e*., all of the vehicles gather data in order to obtain the estimation). Nevertheless, this error value is permissible for the vast majority of ITS applications. Overall, as stated above, both approaches complement each other, allowing us to obtain more accurate and complete density estimations than those obtained by V2I and V2V estimations alone.

### 4.3. Combining V2I and V2V to Estimate Vehicle Density

As stated above, we consider that using approaches based solely on V2V or V2I communications presents some benefits when determining the vehicle density, but these solutions also have some drawbacks. We consider that using the combination of V2I and V2V to determine the density of vehicles provides the benefits of V2V, *i.e*., a microscopic perspective and higher accuracy, along with the benefits of V2I, such as the global density information awareness and the macroscopic vision. In particular, our proposed system makes use of the number of neighbors of each vehicle (V2V), the amount of beacons acquired by each RSU (V2I) and the actual topology to determine the vehicle density.

As a result of the dynamic behavior of the components of our system, static RSUs and moving vehicles make use of different mechanisms to estimate vehicular density. RSUs compute how many beacons are received during a time period, which in our case is set to 30 s, whereas each vehicle computes the average number of neighbors.

**Figure 3:**
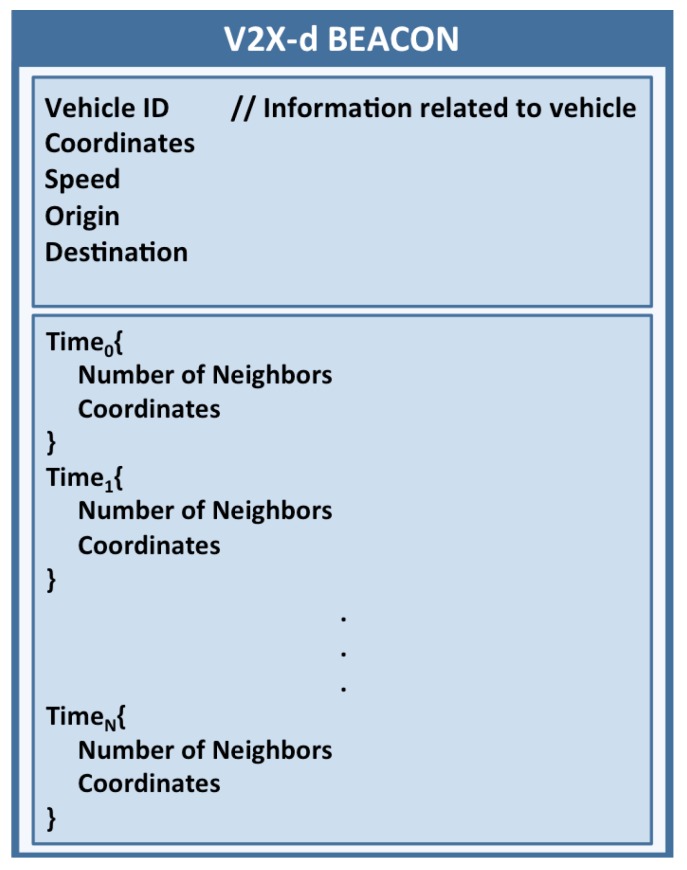
V2X-d beacon structure.

In order to send the information obtained by each vehicle to the control unit, the values estimated by each vehicle are periodically propagated to nearby vehicles by relying on the beacons that are periodically exchanged between vehicles and RSUs. These messages incorporate a field containing the last computed values and the geographic position where the vehicle is located at generation time. Figure 3 shows the proposed beacon format.

With knowledge about the amount of beacons and the mean number of neighbors, both the RSUs and the vehicles themselves are able to precisely predict the instant density of vehicles in their neighborhood. Hence, the control unit in our architecture manages two different values: the vehicle density estimated by RSUs ($d_{V2I}$) and the vehicle density estimated by vehicles ($d_{V2V}$). Then, V2X-d provides the estimated value by calculating the arithmetic mean of these two values to compensate for errors from both sources.

In addition, the V2X-d architecture increases the fault tolerance of the system. For example, if one of the RSUs presents any malfunction, the system will be able to use the information gathered by the vehicles located near that RSU (*i.e*., the vehicles located in its sector). This information would be successfully collected by the control unit when those vehicles reach any other RSU. Then, the control unit could make a more accurate estimation using the estimated values provided by vehicles instead of the values that should be provided by the damaged RSU.

Overall, our proposal will allow supporting different ITS applications by improving the effectiveness of dissemination protocols and reducing network traffic congestion. Additionally, our proposal provides fault tolerance capabilities, since it can continue operating despite any RSU failure.

## 5. V2X-d Performance Evaluation

In this section, we assess the performance of the V2X-d system to prove its accuracy and feasibility. First, we present the simulation environment; then, we assess our proposal under normal conditions; finally, we evaluate it when an RSU fails, proving that our system is still able to estimate the vehicle density under adverse conditions.

### 5.1. Simulation Environment

In order to evaluate our proposal, we have used the ns-2 simulator [40], including some modifications (all of these improvements and modifications are available at [41]) related to the implementation of the 802.11p PHY and MAC layers. In our simulations, nodes operate at the frequency of 5.9 GHz, and all of the vehicles incorporate an IEEE 802.11p interface.

According to Jiang *et al*. [42], we set up 6 Mbit/s as the maximum broadcasting rate in 802.11p and included four different access categories (ACs) that establish the priorities for channel access.

With the purpose of achieving a more uniform coverage area and preventing an unbalanced RSU deployment (*i.e*., too closely or too sparsely), we selected the uniform mesh deployment strategy [34] to deploy RSUs in the map. The uniform mesh strategy reduces the probability of blind areas in the roadmap, *i.e*., areas where vehicles can stay isolated. To ensure the realism of our simulation, we used CityMob for Roadmaps (C4R) [43], a trace mobility engine that directly uses maps obtained from OpenStreetMap [44], thus generating ns-2 compliant traces. Table 6 shows the parameters selected for the simulations.

Table 7 details the topology characteristics of the maps used in our simulations; more specifically, the junctions, the streets, the mean street length and the lanes per street.

In accordance with the results presented in a previous work [25], we included the SJ ratio column, which indicates the relationship between the number of streets and junctions for each scenario. With this metric, it is possible to classify the maps using the SJRatio value: we consider simple topology maps those presenting values lower than one, such as Los Angeles, San Francisco, Madrid, Minnesota and New York. On the contrary, the maps of cities like Rome, Rio de Janeiro, Valencia, Liverpool, Sydney and Amsterdam can be classified as complex (their SJ ratio is higher than one). Complex maps like Rome result in worse communicating results compared to simpler maps, like Minnesota, where the wireless signal easily reaches more vehicles in less time.

**Table 6:** Parameters selected for the simulations. AC, access category.

Parameter	Value
	Rome, Rio de Janeiro, Valencia,
	Liverpool, Sydney, Amsterdam,
roadmaps	Los Angeles, San Francisco,
	Madrid, Minnesota, New York,
roadmap size	2000 m × 2000 m
number of vehicles	[100,200,300…1000]
warning messages priority	AC3
beacon priority	AC1
warning messages size	256 B
beacon size	40 B
message interval	1 s
number of RSUs	9
RSU deployment strategy	Uniform mesh [34]
MAC/PHY	802.11 p
radio propagation model	RAV [45]
mobility model	Krauss [46]
channel bandwidth	6 Mbps
maximum transmission range	400 m

**Table 7:** Map characteristics. SJ, street junction.

Map	Streets	Junctions	Avg.street	Lanes/	SJ Ratio
			Length (m)	Street	
Rome	1655	1193	77.0296	1.0590	1.3873
Rio de Janeiro	542	401	167.9126	1.1135	1.3516
Valencia	2829	2233	60.7434	1.0854	1.2669
Liverpool	1758	1502	361.4686	1.2295	1.1704
Sydney	872	814	138.0716	1.2014	1.0713
Amsterdam	1494	1449	90.8163	1.1145	1.0311
Los Angeles	283	306	408.2493	1.1448	0.9379
San Francisco	725	818	171.4871	1.1749	0.8863
Madrid	628	715	183.4647	1.2696	0.8783
Minnesota	459	591	361.4686	1.0144	0.7766
New York	257	500	489.1328	1.5730	0.5140

### 5.2. Assessing Density Estimation under Normal Conditions

In order to evaluate the accuracy and feasibility of our density estimation proposal, we simulated the roadmap of San Francisco, a scenario with an SJR of 0.8863, considering a density of 150 vehicles per km${}^{2}$ during 30 s. Table 8 shows the beacons received per RSU, the density estimated by each RSU according to this value and the average density estimated. As shown, the V2I subsystem estimates an average density of 137.42 veh./km${}^{2}$, while the V2V subsystem computed an average of 14.16 neighbors per vehicle, resulting in a global estimation of 157.29 veh./km${}^{2}$.

Since we simulated 150 vehicles per km${}^{2}$, in this example, the error obtained in terms of vehicles per km${}^{2}$ by each subsystem separately is of 8.39% and 4.86% for V2I and V2V, respectively. However, using our V2X-d approach, *i.e*., if we combine both subsystems and calculate the average density, we obtain a value of 147.36 veh./km${}^{2}$, which only represents an error of only 1.76%.

As stated above, our system is also able to determine the density distribution in the different zones of the scenario. For instance, we can detect the zones where RSUs acquire a higher number of beacons (see Table 8), which clearly indicates a highly congested zone. According to these results, RSUs 4 and 2 received more beacons than RSUs 3 and 9. Hence, using the V2I communication capabilities, the vehicles’ routes could be changed to those zones where RSUs acquire a lower number of beacons (*i.e*., less congested areas), thereby preventing traffic jams.

**Table 8:** V2I-based estimated vehicle density when simulating 150 vehicles/km${}^{2}$ in San Francisco under normal conditions. RSU, road side unit.

	V2I	
RSU	Normal Situation	
Number	Received	Estimated
	Beacons	veh./km${}^{2}$
1	26	134.24
2	36	185.90
3	6	12.95
4	45	225.31
5	30	156.11
6	28	145.40
7	29	150.81
8	30	156.11
9	16	70.00
**(V2I) Map Density Estimation**		**137.42**

### 5.3. Density Estimation under RSU Failures

In this section, we assess our proposal under adverse conditions, e.g., when an RSU is damaged. The objective is to demonstrate that the V2X-d approach can overcome these situations, providing accurate density estimations. In particular, V2X-d is able to provide alternative estimation values to compensate for the values that the damaged RSU failed to provide to the control unit (V2I subsystem), for those values obtained by the vehicles near the damaged RSU (V2V communications). With this information, the control unit can accurately estimate the vehicle density of the blind zone (*i.e*., near the failing RSU) and thus accurately calculate the density of the whole map.

**Figure 4:**
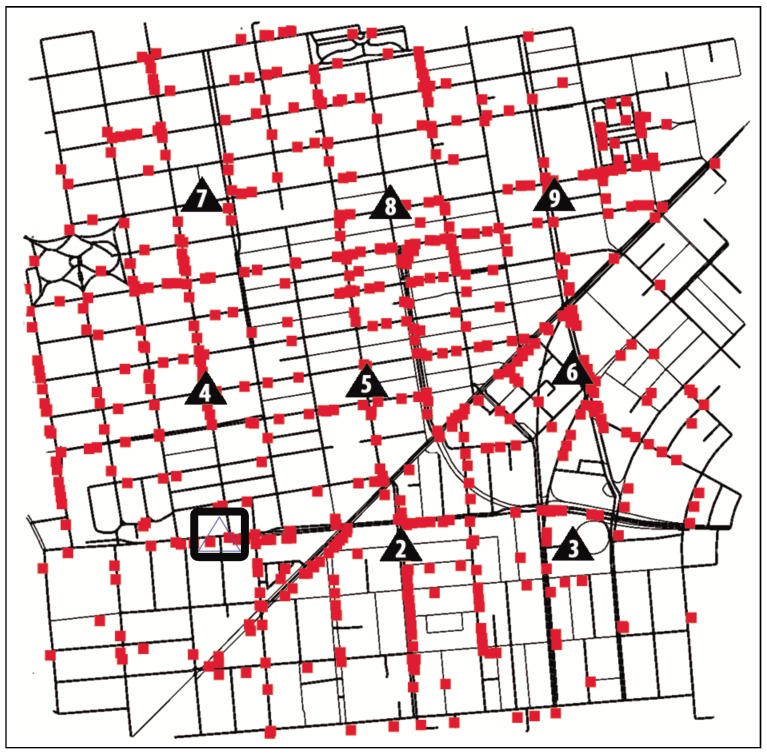
RSUs and vehicle locations in San Francisco when the simulation finished. In this example, RSU 1 is damaged.

To demonstrate the validity of our method, we repeated the experiment presented in Section 5.2, but now considering that RSU 1 presents some problems with respect to estimating the density in its area, thus affecting the global density estimation made by the V2I subsystem (see Figure 4).

To solve this problem, and considering that vehicles store the number of neighbors at different positions along with a timestamp, they would send this information enclosed in their beacons, which would then be received by other RSUs and nearby vehicles. Finally, once the central unit receives the information of the vehicles that have sent data about density in this specific zone, it estimates the density of this area by using the V2V-based estimation function.

In our experiment, the average number of neighbors detected was 11.92, resulting in an estimated density of 132.26 veh/km${}^{2}$. Then, the V2I subsystem estimated that the vehicle density for the target map was 137.2 veh/km${}^{2}$ (see Table 9).

As for the V2V subsystem, it computed an average number of neighbors per vehicle of 14.16, resulting in a density estimation of 157.29 veh./km${}^{2}$. Note that the number of neighbors and the estimated density is not affected by the RSU malfunction.

Based on these data, *i.e*., the density estimated by both the V2I and V2V subsystems, the control unit estimated that the global vehicular density for the target map is 147.25 veh./km${}^{2}$, which only represents an error of 1.83%. This demonstrates that the V2X-d approach can indeed overcome failure situations by completing the missing infrastructure-based information with that provided by nearby vehicles.

**Table 9:** Estimated vehicle density when simulating 150 vehicles/km${}^{2}$ in San Francisco when 1 RSU was broken.

	V2I		V2I	
RSU	1 RSU Damaged		Completed with V2V Information	
Number	Received	Estimated	Received	Estimated
	beacons	Veh./km${}^{2}$	Beacons	Veh./km${}^{2}$
1 (damaged)	0	0	**11.92** (neighbors)	**132.26** (V2V)
2	36	185.90	36	185.90
3	6	12.95	6	12.95
4	45	225.31	45	225.31
5	30	156.11	30	156.11
6	28	145.40	28	145.40
7	29	150.81	29	150.81
8	30	156.11	30	156.11
9	16	70.00	16	70.00
**(V2I) Map Density Estimation**		**122.51**	**Fixed Estimation**	**137.20**

## 6. Conclusions

In this work, we presented V2X-d, a collaborative system able to provide real-time vehicle density estimations in urban scenarios. In particular, our proposal uses smart dynamic sensors and combines V2V and V2I communications. The proposed architecture allows the implementation of better traffic jam mitigation mechanisms, since it facilitates the quick calculation of new routes for vehicles, modifying them according to the particular traffic conditions. In addition, it makes possible the implementation of adaptive broadcast dissemination protocols, improving the communication capabilities in vehicular networks.

The equations used to estimate instant vehicle density were selected after analyzing more than 20 different equations taken from the set provided by ZunZun. In particular, we selected the equations achieving the lowest error (*i.e*., Taylor series for V2I and full cubic for V2V). Based on the knowledge about the amount of beacons received and the average number of neighbors, both the RSUs and the vehicles themselves are capable of quickly estimating the vehicle density. Since the results obtained when modifying the relative weights of V2V and V2I-based estimations are very similar, V2X-d provides the estimated density value by calculating the arithmetic mean of these two values.

Differently from existing approaches, the V2X-d density estimation architecture also takes into account the roadmap topology in the zone where the vehicles are positioned (*i.e*., additionally to the information gathered by vehicles and RSUs). Moreover, it provides resilience capabilities to vehicular networks. In particular, we prove that our approach accurately predicts the density of vehicles, even in adverse environments such as hardware failures in the infrastructure elements. In addition, our approach can robustly overcome the problems raised in other proposals, (*i.e*., caused by adverse weather, inadequate lighting conditions, *etc*.).
